# Postoperative hyperthermia-induced multiple organ failure in a child with Down syndrome: a case report

**DOI:** 10.1186/s13256-022-03305-x

**Published:** 2022-02-27

**Authors:** Keiichi Koizumi, Fuminori Numano, Tomoko Tandou, Ken Takada, Minako Hoshiai, Noboru Oyachi

**Affiliations:** 1 Department of Pediatrics, Fujiyoshida Municipal Hospital, Yamanashi, Japan; 2 Department of Pediatric Surgery, Yamanashi Prefectural Central Hospital, 1-1-1 Fujimi, Kofu, Yamanashi, 400-8506 Japan; 3 Department of Pediatrics, Yamanashi Prefectural Central Hospital, Yamanashi, Japan; 4 Mental Clinic for Child Terra, Yamanashi, Japan

**Keywords:** Child, Down syndrome, Hypercytokinemia, Hyperthermia, Multiple organ failure

## Abstract

**Background:**

Psychological stress has been reported to cause hyperthermia. Persistent excessive hyperthermia can, in turn, cause hypercytokinemia and organ damage. We report a case of postoperative severe hyperthermia leading to a systemic inflammatory response and multiple organ failure in a child with Down syndrome.

**Case presentation:**

A 10-month-old native Japanese boy with Down syndrome and Hirschsprung’s disease is described. Newborn screening showed congenital hypothyroidism and a ventricular septal defect, but these conditions were stable upon administration of levothyroxine and furosemide. His development was equivalent to that of a child with Down syndrome. He developed a noninfectious high fever twice after preoperative preparations at age 8 months and again at 9 months. He was readmitted to hospital at age 10 months to undergo the Soave procedure to correct Hirschsprung’s disease. However, he contracted a fever immediately after the surgical procedure. Hyperthermia (42 °C) was refractory to acetaminophen treatment and deteriorated to multiple organ failure due to hypercytokinemia, with increased serum levels of interleukin-6 (44.6 pg/mL) and interleukin-10 (1010 pg/mL). He died on postoperative day 2 with hypoxemia, respiratory/metabolic acidosis, increased serum levels of transaminases, reduced coagulation, and pancytopenia. Various infectious and noninfectious causes of hyperthermia could not be identified clearly by culture or blood tests.

**Conclusions:**

We speculated that the proximate cause of the fever was psychological stress, because he suffered repeated episodes of hyperthermia after the invasive procedure. Hyperthermia, together with the immune-system disorders associated with Down syndrome, may have induced hypercytokinemia and multiple organ failure. This rare case of noninfectious postoperative hyperthermia leading to multiple organ failure may help to shed further light on the currently unclear pathogenic mechanism of hyperthermia and associated multiple organ failure during the perioperative period in children.

## Background

Excessive psychological stress caused by invasive surgical procedures has been reported to result in hyperthermia via the thermoregulatory center of the hypothalamus [1–3]. Persistent excessive hyperthermia can, in turn, cause organ damage and lead to a poor prognosis [4]. Down syndrome (DS) is also associated with postoperative hyperthermia, and results in overexpression of proinflammatory cytokines as a result of the immune-system disorders occurring in DS [5–7].

Herein, we report a case of postoperative severe hyperthermia leading to a systemic inflammatory response and multiple organ failure (MOF) in a child with DS. This is a rare and interesting case in which prediction of the changes in the clinical course was difficult.

## Case presentation

In July 2019, a native Japanese boy was born at 38 weeks gestation, with body weight of 2846 g and height of 48 cm. He was diagnosed with DS upon chromosomal examination. He had abdominal distension and delayed fecal excretion since birth, and he was referred to our hospital because Hirschsprung’s disease (HD) was suspected. On the 16th day of age, he developed enteritis, and the HD-associated enteritis score was 6 [8]. He was treated with cefmetazole (100 mg/kg/day) and recovered without fever. At 3 months of age, radiography (contrast enema) showed a transition zone 10 cm from the anus. At 4 months of age, anorectal manometry demonstrated the absence of internal relaxation of the anal sphincter. The patient was diagnosed as having short-segment HD. Contrast enema and anorectal manometry did not reveal complications (for example, fever). Newborn screening for inherited diseases of amino acid metabolism, organic acid metabolism, and fatty acid beta-oxidation using tandem mass spectrometry-based microanalysis did not identify a specific disease. Thyroid screening at 2 weeks of age showed hypothyroidism. Hence, levothyroxine (5 μg/kg/day) was administered, and serum levels of thyroid-stimulating hormone and free thyroxine were maintained in the normal range. Echocardiography at birth revealed a ventricular septal defect, and the patient was started on furosemide (1 mg/kg/day). Furosemide was discontinued at 6 months of age because the defective hole shrank spontaneously to 1 mm and there were no findings of pulmonary hypertension or heart failure.

**Fig. 1:**
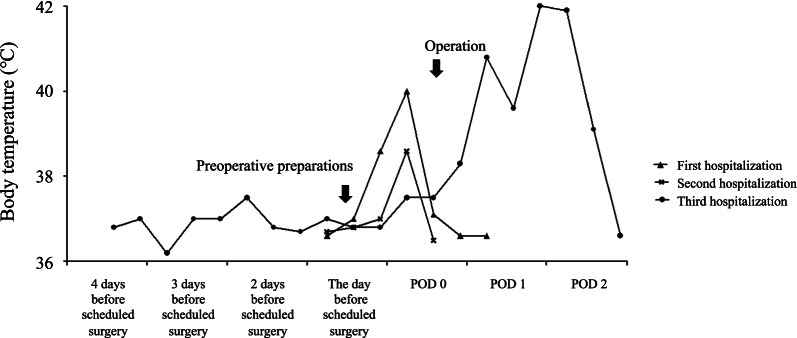
Changes in perioperative body temperature during hospitalization. Each segment on the *X*-axis represents a 24 hour period. The points on the graph represent the body temperature at around 6 am, 2 pm, and 8 pm

**Fig. 2:**
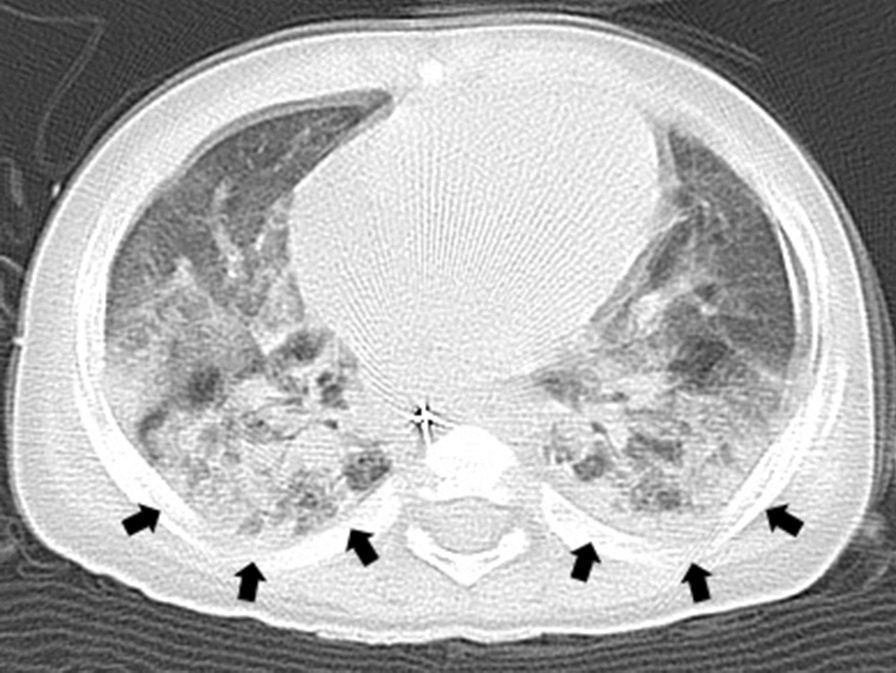
Computed tomography of the lung on postoperative day 1 showing bilateral ground-glass opacities (black arrow)

**Fig. 3:**
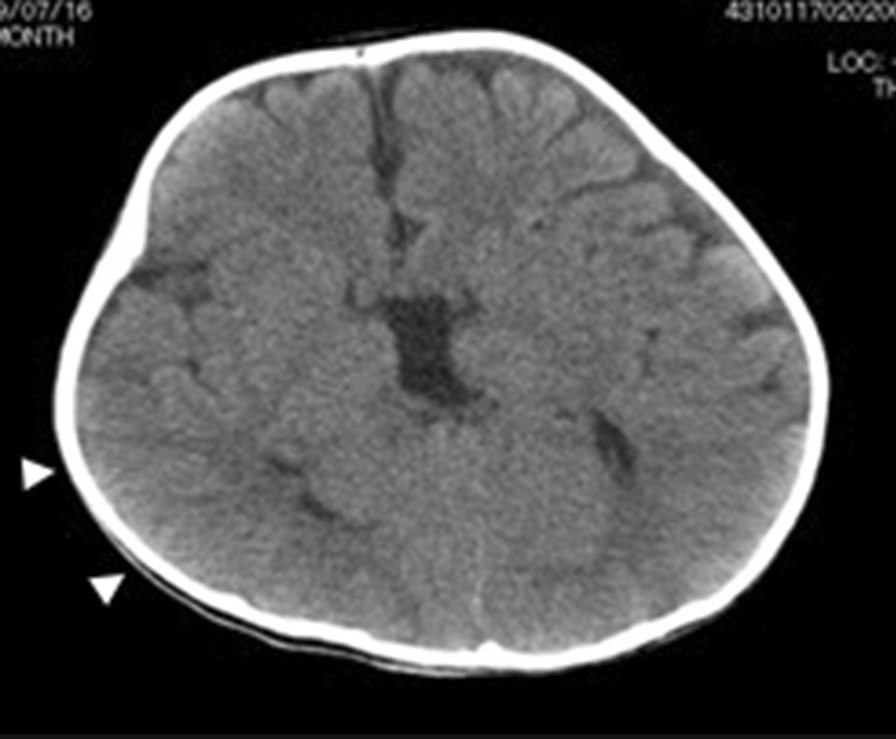
Computed tomography of the brain on postoperative day 1 showing localized cerebral edema with a narrowed sulcus in the right frontal and temporal lobes (white arrow head)

The patient was admitted to hospital at age 8 months and again at 9 months with the aim of carrying out surgery to manage HD. Preoperative blood tests showed that infection parameters (white blood cell count and serum C-reactive protein levels) were in the normal range. Preoperative preparation involved intravenous fluid replacement with fasting and colonic lavage. For the latter, a gastric tube was placed to inject a colon-cleansing agent and a transanal drain was placed to guide the stool. His body movements were restrained to prevent the various medical devices from being removed by himself. However, on the morning of the scheduled surgical procedure, he suddenly developed a body temperature of 38.2–40.0 °C, but physical examination and an absence of symptoms suggested an infectious disease was not present. The procedure was postponed and he was discharged from hospital without medication. His fever resolved immediately the same day (Fig. 1). He was readmitted to hospital for surgery at 10 months of age with body weight of 6.9 kg (−2 standard deviations), height of 63 cm (−2 standard deviations), and Kaup index of 17.3. His development was equivalent to that of a child with DS. The patient was admitted to hospital 4 days before the surgical procedure to observe general status and adapt to the hospital environment. To reduce psychological stress, he was accompanied by his mother throughout the day. During colonic lavage on the day before the surgical procedure, a stool was excreted with a finger instead of transanal drainage. Blood tests on the day before the surgical procedure did not reveal inflammation or fever (Table 1). He underwent the Soave procedure as scheduled. During anesthesia, there was no muscle rigidity, hyperthermia, tachycardia, myoglobinuria, or respiratory/metabolic acidosis suggestive of malignant hyperthermia, and other intraoperative complications were absent. Eight hours after the surgical procedure, his body temperature increased to 40.2 °C and antipyretic medications were ineffective (Fig. 1). He showed no signs of respiratory distress or peripheral circulatory failure, and blood tests did not reveal abnormalities. Fluid replacement was continued, meropenem (120 mg/kg/day)was initiated and the surgical wound dressing was changed. However, he developed status epilepticus on postoperative day (POD) 1, with respiratory failure and a body temperature of 42.0 °C. Mechanical ventilation was initiated. Computed tomography revealed bilateral ground-glass opacities in the lungs, with acute respiratory distress syndrome and localized cerebral edema with partial agenesis of the corpus callosum (Figs. 2, 3). Seizure clusters occurred on POD2 and he was administered midazolam (0.1 mg/kg, intravenous) twice with phenobarbital (20 mg/kg, intravenous) as an anticonvulsant. Despite administration of acetaminophen (15 mg/kg, intravenous), his high fever persisted, together with progressive hypoxemia and respiratory/metabolic acidosis, as well as concurrent increased serum levels of transaminases, reduced coagulation, and pancytopenia (Table 1). He was transferred to the intensive care unit and administered high-dose methylprednisolone (30 mg/kg/day) for suspected acute encephalopathy. His core body temperature was lowered to 36–37 °C by a whole-body cooling system, and hyperosmotic therapy was initiated to treat cerebral edema and catecholamines were given to support cardiac function. However, his condition deteriorated rapidly, with uncontrolled bradycardia and hypotension. Multiple organ dysfunction occurred and the patient died on POD2.

**Table 1:** Blood and cerebrospinal fluid examination at the time of the third hospitalization

Blood examination						
		The day before surgery	POD 0	POD 1	POD 2	Reference range
WBC	(/μL)	8700	10700	8400	5300	6000–17500
Hemoglobin	(g/dL)	14.1	10.9	9.2	7.0	10.5–14.1
Platelet	(10^3^/μL)	450	307	207	16	150–400
TP	(g/dL)				3.6	5.9–7.5
Albumin	(g/dL)				2.9	3.4–4.7
T-bil	(mg/dL)	6.3	4.1	4.5	0.7	0.2–0.7
AST	(U/L)	4.4	2.8	3.0	2730	23–51
ALT	(U/L)	0.3	0.4	0.3	2206	5–25
LDH	(U/L)	48	51	73	3523	202–437
CPK	(U/L)	24	17	23	2959	54–389
BUN	(mg/dL)	256	234	394	17.0	6–20
Creatinine	(mg/dL)	143	676	1013	0.91	0.14–0.34
Na	(mEq/L)	10.2	5.6	6.4	147	139–146
K	(mEq/L)	0.33	0.49	0.47	3.9	4.1–5.3
Cl	(mEq/L)	140	143	141	114	98–106
CRP	(mg/dL)	4.8	3.6	3.3	2.49	< 0.14
Lactate	(mg/dL)	105	109	109	62	3–17
TSH	(μIU/mL)	0.02	0.42	6.75	1.25	0.42–4.3
Free triiodothyronine	(ng/mL)		11	27	3.3	2.28–4.56
Free thyroxine	(ng/mL)				1.9	0.99–1.91
Cortisol	(μg/mL)				27	3–23
PT-INR				1.40	3.66	0.75–1.15
APTT	(sec)	0.85		49	251	30–45
Fibrinogen	(mg/dL)	34		308	105	200–400
D-dimer	(μg/mL)	266		2.8	7.8	0.15–1.0
pH			7.311	7.454	7.225	7.35–7.45
pCO_2_	(mmHg)		43.8	27.5	23.9	35–45
pO_2_	(mmHg)				80.3	80–100
HCO_3_^−^	(mmol/L)		21.4	19.0	9.5	22–26
BE	(mmol/L)		−4.1	−3.6	−16.6	0 ± 2
IL-6	(pg/mL)	1.2			44.8	< 4.0
IL-10	(pg/mL)	< 2			1010	< 5.0
TNF-α	(pg/mL)	0.7			0.6	< 1.66

Cerebrospinal fluid examination						
		The day before surgery	POD 0	POD 1	POD 2	Reference range
WBC	(/μL) (pg/mL)				1	0–6
IL-6					4.4	< 19.9
IL-10	(pg/mL)				< 2	< 14.2
TNF-α	(pg/mL)				< 0.15	< 11.1

*ALT* alanine aminotransferase, *APTT* activated partial thromboplastin time, *AST* aspartate aminotransferase, *BE* base excess, *BUN* blood urea nitrogen, *CPK* creatine phosphokinase, *CRP* C-reactive protein, *IL* interleukin, *LDH* lactate dehydrogenase, *POD* postoperative day, *PT-INR* prothrombin time-international normalized ratio, *T-bil* total bilirubin, *TNF* tumor necrosis factor, *TP* total protein, *TSH* thyroid-stimulating hormone, *WBC* white blood cell

We assessed the results of examinations on POD2. Blood, cerebrospinal fluid, and urine cultures were negative. The FilmArray Respiratory Panel (a multiplex real-time polymerase chain reaction) did not detect respiratory pathogens. Analyses of cerebrospinal fluid showed a normal white blood cell count and normal levels of cytokines. Blood tests showed normal thyroid and adrenal functions (Table 1). Electroencephalography revealed no specific etiologies associated with encephalopathy. Furthermore, there were no clinical signs of infection (for example, otitis media, urinary tract) during the postoperative course and no postoperative complications (for example, necrotic soft-tissue infection or anastomotic leakage) on abdominal ultrasonography or radiography. Family history did not include epidemic infections or hereditary diseases. His mother was a nurse and he lived in an affluent and hygienic environment.

Considering the clinical course and newborn screening, we excluded respiratory infection, bacteremia, meningitis, encephalopathy, malignant hyperthermia, and hyperthyroidism, as well as inherited genetic and metabolic disorders. However, his death was associated with hypercytokinemia, with increased serum levels of interleukin (IL)-6 and IL-10 on POD2 compared with those before surgery.

## Discussion

Our patient experienced repeated episodes of hyperthermia during the perioperative period, including severe postoperative pyrexia leading to hypercytokinemia, which, in turn, progressed to MOF. Pediatric neurologists, pediatric surgeons, and pediatric intensivists were in charge of his care, but the specific causes of his hyperthermia, hypercytokinemia, and MOF were not identified clearly.

It has been reported that 80% of the causes of fever in the early postoperative period are noninfectious, such as necrotizing soft-tissue infection, pulmonary embolism, anastomotic leak, adrenal insufficiency, and malignant hyperthermia [9]. In this case, infection, postoperative complications, and metabolic/endocrine disorders could be excluded as causes of fever. Stress-induced hyperthermia was considered the most probable cause based on the clinical course and literature review.

Psychological stress increases the body temperature via the thermoregulatory center of the hypothalamus, as well as by activation of the central and sympathetic nervous systems. This pathophysiology is independent of the cascade of proinflammatory cytokines and prostaglandin E_2_, so suppression by nonsteroidal antiinflammatory drugs is not possible [1–3]. Several case reports of stress-induced hyperthermia in the perioperative period have been published, but progression to MOF has not been reported [10, 11]. The degree of perioperative stimulation required to activate a systemic immune response varies among individuals because of differences in genetic predisposition/susceptibilities [12]. Patients with DS and cognitive dysfunction have been reported to be more sensitive and much less resilient to psychological stress [13]. Psychogenic fever has been reported to account for 18% of cases of fever of unknown origin in children. It has been suggested that changes in the autonomic nervous system and hormone secretion or psychological instability in children may contribute to the cause of stress [14]. In this case, these stresses may have induced severe hyperthermia three times during the perioperative period that was unresponsive to antipyretic treatment.

Although the pathogenesis of stress-induced hyperthermia does not involve a “cytokine storm,” the hyperthermia itself can affect expression of cytokines such as IL-1, tumor necrosis factor (TNF)-α, IL-6, and IL-10 [1, 3, 15]. A prolonged period with a body temperature > 40 °C is associated with a worse outcome, and such hyperthermia can lead to irreversible MOF and death. Even if cooling procedures and intensive care management are started promptly, these cannot halt the course of worsening MOF [4, 15]. Furthermore, DS pathogenesis involves abnormally high numbers of activated cluster of differentiation (CD)14^+^CD16^+^ monocytes, which overexpress proinflammatory cytokines [5–7]. We speculated that the hypercytokinemia, systemic inflammatory response syndrome, and MOF observed in our patient were caused by hyperthermia and the immune system abnormalities associated with DS. The increased serum levels of IL-6 and IL-10 immediately postoperatively were as high as documented in previous reports of influenza-associated encephalopathy in hypercytokinemia [16, 17]. Cytokine release from inflammatory cells may have led to the development of acute respiratory distress syndrome postoperatively.

IL-10 is an antiinflammatory cytokine that inhibits the release and production of TNF-α and IL-6 by monocytes. IL-10 overproduction has been positively correlated with mortality, and a high IL-10:TNF-α ratio is associated with a fatal outcome in cases with an early-stage systemic inflammatory reaction [17, 18]. In our patient, low serum levels of TNF-α and high serum levels of IL-10 were related to the fatal outcome.

Anxiolytic drugs (for example, benzodiazepines and agonists of serotonin 1A receptors) attenuate stress-induced hyperthermia by inhibiting the central and sympathetic nervous systems [1, 3]. Our patient was administered benzodiazepines as an anticonvulsant on POD2. However, we suspected that MOF had already progressed at that time, so benzodiazepines failed to exert an antipyretic effect. We suggest that plasmapheresis, which can inhibit activation of CD14^+^CD16^+^ monocytes and hypercytokinemia, may be useful in patients at this stage of MOF [19, 20].

Metabolic disorders cannot be excluded by screening using tandem mass spectrometry only at birth and may progress. Even if bacteria or viruses are not detected, an infection may have developed. We could not clarify why the stress experienced in preoperative HD-related enteritis (and tested using contrast enema and anorectal manometry) did not induce fever.

## Conclusions

We have described a very rare and interesting case of noninfectious postoperative hyperthermia leading to MOF. This case report provides valuable information regarding the pathogenic mechanism of hyperthermia and associated MOF (especially during the perioperative period) in children. Additionally, psychological stress can cause unpredictable phenomena in children with DS, so great care should be taken when undertaking invasive examinations and surgical procedures in this population.

## Data Availability

Not applicable.

## Abbreviations

DS: Down syndrome
HD: Hirschsprung’s disease
IL: Interleukin
MOF: Multiple organ failure
POD: Postoperative day
TNF: Tumor necrosis factor
